# Impact of Positioning Errors on the Dosimetry of Breath-Hold-Based Volumetric Arc Modulated and Tangential Field-in-Field Left-Sided Breast Treatments

**DOI:** 10.3389/fonc.2020.554131

**Published:** 2020-10-29

**Authors:** Yanqun Zhao, Peng Diao, Da Zhang, Juxiang Wu, Xin Xin, Davide Fontanarosa, Min Liu, Jie Li, Lucia Clara Orlandini

**Affiliations:** ^1^ Department of Radiation Oncology, Sichuan Cancer Hospital and Research Institute, Chengdu, China; ^2^ School of Medicine, University of Electronic Science and Technology of China, Chengdu, China; ^3^ Radiation Oncology Key Laboratory of Sichuan Province, Chengdu, China; ^4^ School of Clinical Sciences, Queensland University of Technology, Brisbane, QLD, Australia; ^5^ Institute of Health & Biomedical Innovation, Queensland University of Technology, Brisbane, QLD, Australia

**Keywords:** ****left-sided breast, radiotherapy, volumetric modulated radiation therapy, breath hold, tangential field

## Abstract

Heart diseases and cardiovascular events are well-known side effects in left-sided breast irradiation. Deep inspiration breath hold (BH) combined with fast delivery techniques such as volumetric modulated arc therapy (VMAT) or tangential field-in-field (TFiF) can serve as a valuable solution to reduce the dose to the heart. This study aims to compare the impact of positioning errors in VMAT and TFiF plans for BH left-sided breast treatments. Fifteen left-sided breast patients treated in BH with TFiF technique were included in this retrospective study. For each patient, a second plan with VMAT technique was optimized. Eighteen setup variations were introduced in each of these VMAT and TFiF reference plans, shifting the isocenter along six different directions by 3, 5, and 10 mm. A total of 540 perturbed plans, 270 for each technique, were recalculated and analyzed. The dose distributions on the target and organs at risk obtained in the different perturbed scenarios were compared with the reference scenarios, using as dosimetric endpoints the dose-volume histograms (DVH). The results were compared using the Wilcoxon test. Comparable plan quality was obtained for the reference VMAT and TFiF plans, except for low doses to organs at risk for which higher values (p < 0.05) were obtained for VMAT plans. For TFiF plans, perturbations of the isocenter position of 3, 5, or 10 mm produced mean deviations of the target DVH dosimetric parameters up to −0.5, −1.0, and −5.2%, respectively; VMAT plans were more sensitive to positioning errors resulting in mean deviations up to −0.5, −4.9, and −13.9%, respectively, for the same magnitude of the above mentioned perturbations. For organs at risk, only perturbations along the left, posterior, and inferior directions resulted in dose increase with a maximum deviation of +2% in the DVH dosimetric parameters. A notable exception were low doses to the left lung and heart for 10 mm isocenter shifts for which the mean differences ranged between +2.7 and +4.1%. Objective information on how external stresses affect the dosimetry of the treatment is the first step towards personalized radiotherapy.

## Introduction

Breast cancer is the most common cancer in women worldwide. The standard of care includes conservative surgery or mastectomy as appropriate, followed by adjuvant radiotherapy. Radiotherapy is essential to improve local tumor control and overall survival; nevertheless, delivery of some dose to heart, lungs, and contralateral breast is unavoidable. Increased risk of fatal cardiac events, pneumonitis, or a second primary cancer of the breast has been largely reported (1–4). Given the occurrence of this disease even at a young age and the increased life expectancy, it is essential to limit as much as possible long-term complications.

Deep inspiration breath-hold (DIBH) in left-sided breast treatment increases the distance between the target and the heart as well as the part of lungs included in the treatment field. Many studies have been published on the benefits of DIBH showing how it enables minimized irradiation of nearby organs at risk while maintaining an adequate target dose coverage (5–8), and, therefore, has become the gold standard in clinical practice in many institutions (9–12).

Breast cancer treatment has historically been performed with two opposing non-divergent isocentric tangent fields, using six MV photon energies, with the addition of beam modifiers to homogenize the dose within the target (13, 14). The tangential field-in-field technique (TFiF), also referred to as forward intensity modulated radiation therapy, can be used as an alternative (15, 16); it consists of two open opposing tangential fields, but instead of wedges for target dose homogenization, additional fields (usually two to four) are manually created using a multileaf collimator (MLC). The main field and the subfields are therefore merged into one beam that includes several segments for the sequential irradiation. However, high intensity modulated radiotherapy techniques, such as volumetric modulated arc therapy (VMAT) and intensity modulated radiotherapy can achieve more homogeneous dose distribution within complex targets, such as concave-shaped breasts (17, 18). Moreover, inverse planning modalities have the advantage of optimizing the plan based on the clinical goals, offer fast planning options, and allow more tailored doses to organs at risk (OARs) (19, 20). Additionally, VMAT provides fast treatment delivery and is therefore an optimal candidate to pair with DIBH.

Others authors investigated the use of VMAT in the breath-hold treatment of left-sided breast radiotherapy and found unquestionable advantages for the heart dosimetry (21, 22). A paramount aspect to consider in these treatments is the uncertainty in inter-fraction patient re-positioning, which can lead to inaccuracies in the administered dose (23); when positioning errors occur, the steepness of the dose-effect curves can limit the effectiveness of highly modulated techniques such as VMAT, thus influencing the patient’s results both for local tumor control and for normal tissue complications. Moreover, daily shifts in patient setup are inevitable even with image guidance (24), and since it has become increasingly more evident in literature that imaging dose delivered to patients for pre-treatment image verification with cone-beam computed tomography (25), can be a concern (26), it is often not scheduled on a daily basis.

This work focuses on the use of BH with conformal TFiF and highly conformal VMAT irradiation techniques. Investigates whether, with an increased distance of the heart and lung from the target, VMAT and TFiF plans are robust against isocenter positioning errors; and it evaluates quantitatively their dosimetric impact on treatment plans for different magnitudes and directions.

## Materials and Methods

This retrospective study included fifteen female early-stage pT1c-T2N1 left breast carcinoma patients receiving radiation therapy in DIBH between April and August 2019, after a breast-conserving surgery. The anatomical and clinical characteristics of the patients enrolled in this study are reported in **Table 1**. The study was reviewed and approved by the Ethics Committee of our Hospital (approval number SCCHEC 02-2020003).

**Table 1:** Patient characteristics.

Patient #	Age*	Patient anatomy				Clinical characteristics	
		PTV volume (cm^3^)	Heart volume (cm^3^)	Lung volume^ (cm^3^)	Body mass index (kg/m^2^)	Grade	pTNM
1	47	441.5	360.2	3571.5	19.7	G3	T1cN0M0
2	23	88.3	408.2	4543.6	22.2	G2	T1cN0M0
3	31	509.8	422.4	2356.4	20.8	G2	T1cN0M0
4	49	383.0	637.5	3701.6	17.4	G1	T1cN0M0
5	49	486.7	429.5	4150.9	18.8	G1	T2N0M0
6	34	598.6	469.9	2399.9	21.9	G3	T1cN0M0
7	39	500.8	518.8	3963.7	21.8	G2	T1cN0M0
8	31	638.3	540.9	4022.7	20.8	G2	T1cN0M0
9	56	389.9	595.9	3146.3	25.0	G1	T2N0M0
10	33	247.5	447.1	3112.0	19.6	G1	T1cN0M0
11	48	484.7	459.2	3627.1	20.5	G2	T1cN0M0
12	44	654.4	342.3	4473.2	21.0	G2	T1cN0M0
13	55	497.8	577.2	3625.5	19.8	G2	T1cN0M0
14	33	776.2	544.5	4236.4	21.3	G3	T1cN1M0
15	58	461.0	575.6	3492.8	19.2	G1	T1aN0M0
Mean	42	510.5	488.6	3628.2	20.7	–	–
Median	44	497.8	469.9	3627.1	20.8	–	–

PTV, planning target volume; pTNM, pathological tumor node metastasis stage; *at the time of radiotherapy; ^volumes at breath hold setup.

### Clinical Workflow

In DIBH treatment, patients must be able to inspire and then maintain inspiration during each treatment. At the planning computed tomography (CT), time was dedicated to patient training. Patients were immobilized in a supine position with a standard commercial breast board (WingSTEP, IT-V, Innsbruck, Austria) that allowed them to comfortably rest their arms behind their heads; an anterior and two lateral tattoos were performed at the free breathing setup position corresponding to the laser cross. Patients were then asked to inhale so as to swell the upper chest and to hold their breath, and three additional skin marks were performed at the laser cross to set the breathing retention setup. To be eligible for DIBH treatment, patients must be able to hold their breath for at least 25 s and to replicate the breath retention setting five times in succession. The setup reproducibility at the CT training was verified by checking the alignment of tattoos/lasers in the free breathing setup, followed by the alignment of marks/lasers in breath hold; moreover, the height of the breath-hold lateral tattoos above the couch top during breath-hold were registered and compared with the height of the lateral tattoos on the CT scan to confirm that a consistent breath-hold was performed. CT scans with 3 mm slice thickness were acquired in breath-hold with a 16-slice Brilliance Big Bore CT (Philips Medical Systems, Cleveland, OH). In the treatment room, the patient is first aligned in free breathing; then, in the same way as at the CT training session, during the breath-hold retention the accuracy and repeatability of the breath-hold setup is verified and, for each treatment field, the field border is marked on the patient skin. The patient was asked to perform a breath-hold; using treatment room cameras, therapists at the LINAC’s console administered the radiation when the light field and previously marked field borders were correlated. Electronic portal images acquired for each beam in breath-hold before delivery were matched online to digitally reconstructed radiographs. The voluntary breath-hold technique described above has been implemented and used in the clinical practice by the Royal Marsden Hospital in London (UK) (27). Barlett et al. (28) found comparable results in the reproducibility and normal tissue sparing of this technique and that of ABC (active breathing coordinator); however, they noted that patients found the voluntary DIBH technique more comfortable and less claustrophobic.

Experienced radiation oncologists from the breast oncology department outlined the target and the OARs on the CT images dataset imported into Pinnacle 3TM Version 9.10 (Philips Medical Systems, Eindhoven, the Netherlands). The clinical target volume (CTV) consisted of the whole left breast, excluding pectoralis muscles, chest wall muscles, and ribs. The planning target volume (PTV) was an isotropic expansion of the CTV with a 3 mm margin in all directions; the first 5 mm inside the body external contour were excluded both from the CTV and from the PTV. OAR delineations were performed according to the breast cancer atlas for the radiation therapy planning consensus definitions (29). An hypo-fractionated regimen as standard of care for early stage breast cancer (30) has been adopted in our center with a prescribed dose (D_p_) of 42.56 Gy delivered in 16 fractions (31, 32) over 3 weeks. The plan has been optimized to achieve minimum 95% of the PTV covered by 95% isodose line and a mean dose to PTV equal to the D_p_; hotspots should not exceed 107%, although they were considered acceptable if 2 cm^3^ of the target received 110% of the D_p_. For OARs standard dose limits were used (33, 34) aiming at keeping a heart D_m_ under 2 Gy and less than 15% of the left lung receiving less than 20 Gy (10).

### Tangential Field in Field and Volumetric Modulated Arc Therapy Reference Plans

An Elekta Infinity (Elekta, Stockholm, Sweden) LINAC mounting a 5 mm MLC was used for the treatments. The TFiF treatment plan delivered to the patients included in this study was performed with a 6 MV beam and consist of two opposing tangential fields with gantry angle between 300° and 315° for the medial beam and 120° and 135° for the lateral beam, each including two or three sub-segments. The main segment that corresponds to the whole tangential field consisted of about 80% of the monitor units (MU). TFiF is a forward plan and its clinical implementation is closely related to conventional planning. Manual definition of the segments leads to intuitive choices for the segment shapes on the beam’s-eye-view option of the planning system. Two opposite open tangential fields were initially created, equal weights were assigned and the corresponding dose distribution was calculated. High-spot volumes were created from the isodoses, and subfield of the tangential beams were added manually conforming the leaves of the MLC to cover the hot-spot volumes; the dose distribution was then recalculated and the weights of field and subfields were adjusted to improve the dose homogeneity. This process was repeated until the accurate dose distribution was reached. The number of subfields varied between two and three. The plan was constrained to a delivery time for each beam shorter than 20 s to introduce a safety margin with respect to the inclusion criteria. The plan was calculated using the full collapsed cone convolution algorithm and a grid calculation size of 3 mm. The TFiF plan clinically delivered, represents the TFiF reference plan (TFiF_ref_) for the purpose of this research.

For each case, a second plan was retrospectively implemented for the purpose of our research, using the VMAT technique and the same LINAC but with the 6 MV flat flattening free beam energy and dose rate of 1,400 MU/min. This choice was made to guarantee the shortest delivery time, which is advantageous for a patient treated in breath hold, with a target metric comparable to the one obtainable with flattened filter beams (35–37); we will refer to it as VMAT reference plan (VMAT_ref_). The plan consisted of two 40° partial arcs with 300°/340° and 100°/140° as start/stop angles, respectively, with a variability of 10° in the start/stop gantry angles based on the specifics of the patient’s treatment plan; the plan was optimized following the same criteria for the maximum delivery time, OARs dose constraints, target dose coverage. The same radiation oncologist approved the TFiF_ref_ and, successively, the VMAT_ref_ plans.

### Plans Perturbations Comparisons

Eighteen setup variations were introduced on each VMAT_ref_ and TFiF_ref_ plan, shifting the isocenter from its reference position in the superior (S), inferior (I), left (L), right (R), anterior (A), and posterior (P) directions with respect to the patient couch view from the feet of the patient by 3, 5, and 10 mm. A total of 540 treatment plans were recalculated with these simulated positioning errors on the planning CT without changing any parameter other than the position of the isocenter. Dose volume histogram (DVH) endpoints were used to compare the impact of the variations in setup on the dosimetry of the VMAT and TFiF plans; in particular, D_95_, D_98_, D_2cc_, and D_m_ for the CTV; V_5_, V_10_, V_25_, and D_m_ for the heart; V_5_, V_20_, and D_m_ for the left lung; and V_5_ and D_m_ for the right breast, were used, where D_x_ represented the dose (in Gy) received by x% of the volume, V_y_ the volume (in percentage) receiving y Gy, and D_2cc_ the dose in Gy received by a volume of 2 cm^2^. Absolute differences ΔD_x_, ΔV_y_, and ΔD_m_ were calculated by subtracting the reference value D*_x_*, V*_y_*, D_m_, respectively, from the corresponding perturbed value.

Datasets were compared using the Wilcoxon signed-rank test. A *p* value of less than 0.05 was considered statistically significant; IBM SPSS v20 software (IBM, Armonk, US) was used for the analysis.

## Results

CTV and OARs DVH dosimetric parameters (mean and range values), obtained for the non-perturbed reference VMAT and TFiF plans, are shown in **Table 2**. Target coverage was clinically acceptable for both techniques and for all the cases studied, with a minimum D_95_ value of 40.4 Gy, D_m_ equal to D_p_ ± 0.3 Gy, and a maximum D_2cc_ value of 45.4 Gy. Similarly, standard dose constraints were satisfied for all OARs; heart D_m_ resulted <2 Gy, with the exception of one case that achieved a D_m_ of 3.2 Gy; and left lung V_20_ < 15% with a maximum value registered of V_20_ = 16.4%. D_m_ for the right breast was <2.4 Gy with a mean value of 1.5 Gy.

**Table 2:** Dose-volume histograms (DVH) dosimetric parameters mean and range for volumetric modulated arc therapy (VMAT) and tangential field in field (TFiF) reference (non-perturbed) plans of the 15 patients; in bold *p* values < 0.05.

		VMAT	TFiF	*p* value
CTV	D_95_ (Gy)	41.0 (40.4–41.4)	40.9 (40.4–41.4)	0.535
	D_98_ (Gy)	40.3 (39.9–40.7)	40.3 (39.6–40.4)	0.430
	D_2cc_ (Gy)	43.9 (43.3–45.5)	44.4 (43.4–45.4)	0.327
	D_m_ (Gy)	42.6 (42.3–42.8)	42.8 (42.5–42.9)	0.342
Heart	V_5_ (%)	4.2 (3.8–6.0)	3.1 (1.1–5.2)	**0.052**
	V_10_ (%)	1.2 (0.6–2.6)	1.3 (0.4–2.5)	0.061
	V_25_ (%)	0.2 (0.0–0.4)	0.3 (0.0–0.8)	0.370
	D_m_ (Gy)	1.6 (0.5–3.2)	1.5 (0.5–2.9)	0.350
Left lung	V_5_ (%)	29.0 (23.2–34.5)	25.7 (21.0–30.0)	**0.037**
	V_10_ (%)	20.0 (15,3–24.8)	19.2 (15.3–22.3)	0.132
	V_20_ (%)	12.4 (9.1–16.4)	12.8 (9.2–16.4)	0.230
	D_m_ (Gy)	6.6 (5.3–8.2)	6.6 (5.3–7.9)	0.123
Right breast	V_5_ (%)	1.7 (0.2–3.0)	1.1 (0.0–2.2)	**0.034**
	D_m_ (Gy)	1.5 (0.8–2.4)	1.5 (0.5–2.4)	0.321

DVH, dose volume histogram; VMAT, volumetric modulated arc therapy; TFiF, tangential field in field; CTV, clinical target volume; D_x_, dose in Gy received by x% of the volume; D_m_, mean dose; V_x_, volume receiving x Gy; D_2cc_, dose in Gy received by a volume of 2 cube centimeters.

The comparison of the target coverage obtained with the reference *vs*. the perturbed plans is shown in **Table 3**; the mean and range of the absolute differences of CTV DVH dosimetric parameters obtained are reported for different magnitudes and directions of the isocenter shifts.

**Table 3:** Mean value and range of clinical target volume (CTV) dose volume histogram (DVH) dosimetric parameters absolute difference between the reference and perturbed volumetric modulated arc therapy (VMAT) and tangential field in field (TFiF) plans for different isocenter shifts.

Isocenter shift Dir; mm	CTV, ΔD_95_ (Gy)		CTV, ΔD_98_ (Gy)		CTV, ΔD_m_ (Gy)	
	TFiF	VMAT	TFiF	VMAT	TFiF	VMAT
I; 3	−0.1 (−0.1; −0.3)	−0.1 (0.0; −0.1)	−0.1 (0.0; −0.3)	−0.1 (0.0; −0.2)	0.0 (0.0; −0.1)	−0.1 (0.0; −0.3)
S; 3	−0.2 (−0.1; −0.4)	−0.2 (−0.1; −0.3)	− 0.2 (−0.1; −0.4)	−0.2 (−0.1; −0.4)	−0.2 (−0.1; −0.5)	−0.2 (−0.1; −0.4)
L; 3	−0.2 (−0.1; −0.4)	−0.3 (−0.3; −0.4)	−0.2 (−0.1; −0.3)	−0.3 (−0.2; −0.5)	−0.2 (−0.1; −0.4)	−0.1 (0.0; −0.3)
R; 3	−0.2 (0.0; −0.3)	−0.2 (−0.1; −0.4)	−0.2 (0.0; −0.3)	−0.3 (−0.1; −0.6)	−0.2 (0.0; −0.3)	−0.2 (−0.1; −0.3)
P; 3	−0.1 (−0.1; −0.2)	−0.1 (0.0; −0.2)	−0.1 (0.0; −0.1)	−0.1 (−0.1; −0.2)	−0.1 (0.0; −0.2)	−0.2 (−0.2; −0.3)
A; 3	−0.3 (−0.2; −0.5)	−0.4 (−0.2; −0.6)	−0.4 (−0.2; −0.6)	−0.4 (−0.3; −0.5)	−0.3 (−0.1; −0.4)	−0.3 (−0.1; −0.3)
All Dir	−0.2 (−0.0; −0.5)	−0.2 (−0.0; −0.6)	−0.2 (0.0: −0.6)	−0.2 (0.0: −0.6)	−0.2 (0.0; −0.5)	−0.2 (0.0; −0.4)
I; 5	−0.2 (−0.1; −0.3)	−0.8 (−0.4; −1.5)	−0.2 (0.0; −0.2)	−1.0 (−0.6; −1.4)	−0.1 (0.0; −0.3)	−0.3 (−0.1; −0.3)
S; 5	−0.3 (−0.1; −0.4)	**−1.7 (−1.1; −2.7)**	−0.4 (−0.2; −0.6)	**−2.2 (−2.0; −2.7)**	−0.2 (−0.1; −0.4)	−0.2 (−0.1; −0.4)
L; 5	−0.3 (−0.2; −0.4)	−0.9 (−0.5; −1.9)	−0.3 (−0.2; −0.4)	−1.1 (−0.7; −1.5)	−0.2 (−0.1; −0.4)	−0.3 (−0.1; −0.4)
R; 5	−0.4 (−0.2; −0.5)	**−2.1 (−1.9; −2.7)**	−0.4 (−0.2; −0.6)	**−2.9 (−2.0; −3.6)**	−0.2 (−0.1; −0.3)	−0.2 (0.0; −0.3)
P; 5	−0.1 (0.0; −0.3)	−1.0 (−0.8; −1.6)	−0.2 (−0.2; −0.3)	**−1.6 (−1.2; −1.8)**	−0.1 (0.0; −0.2)	−0.1 (0.0; −0.3)
A; 5	−0.5 (−0.4; −0.8)	**−2.6 (−2.2; −3.0)**	−0.6 (−0.4; −1.4)	**−3.2 (−2.4; −4.5)**	−0.3 (−0.2; −0.5)	−0.3 (−0.2; −0.5)
All dir	−0.3 (−0.1; −0.8)	**−1.5 (−0.5; −2.9)**	−0.4 (0.0: −1.4)	**−2.0 (−0.6; 4.5)**	−0.2 (0.0; −0.5)	−0.2 (−0.1; −0.5)
I; 10	−0.5 (−0.1; −0.6)	**−1.4 (−1.2; −1.7)**	−1.1 (−0.7; −1.4)	**−2.7 (−2.2; −3.4)**	−0.3 (−0.3; −0.4)	−0.9 (−0.4; −1.1)
S; 10	**−1.1 (−0.9; −1.3)**	**−3.4 (−3.0; −3.6)**	**−2.0 (−1.3; −3.0)**	**−5.3 (−4.5; −6.1)**	−0.5 (−0.4; −0.9)	−0.8 (−0.4; −1.1)
L; 10	−0.7 (−0.6; −1.0)	**−2.1 (−1.6; −2.4)**	−0.9 (−0.7; −1.3)	**−3.2 (−2.1; −4.3)**	−0.4 (−0.2; −0.6)	−0.9 (−0.9; −1.3)
R; 10	−2.2 (−1.9; −2.6)	**−6.3 (−5.7; −6.3)**	**−3.2 (−2.8; −3.8)**	**−8.9 (−6.9; −10.1)**	−0.6 (−0.4; −0.8)	−1.3 (−0.8; −1.1)
P; 10	−0.7 (−0.4; −1.6)	**−2.0 (−1.6; −2.2)**	−1.2 (−0.6; −1.8)	**−3.4 (−2.9; −3.9)**	−0.2 (−0.1; −0.3)	−0.9 (−0.7; −1.0)
A; 10	**−2.6 (−2.0; −3.3)**	**−7.3 (−5.3; −8.1)**	**−3.8 (−3.5; −4.3)**	**−10.1 (−6.5; −12.1)**	−0.8 (−0.5; −1.1)	−1.2 (−0.7; −1.4)
All dir	**−1.3 (−0.1; −3.3)**	**−3.8 (−1.2; −8.1)**	**−2.1 (−0.6; −4.3)**	**−5.6 (−2.1; −12.1)**	−0.5 (−0.1; −1.1)	−1.0 (−0.4; −1.4)

VMAT, volumetric modulated arc therapy; TFiF, tangential field in field; CTV, clinical target volume; D_x_, dose in Gy received by x% of the volume; D_m_, mean dose; V_x_, volume receiving x Gy. I, inferior; S, superior; P, posterior; L, left; R, right; A, anterior; P. posterior; Dir, direction.

In gray data that with mean absolute difference ≤2%, in bold > 3%.

For a 3 mm perturbation, both techniques showed a mean absolute difference ΔD_95_ and ΔD_98_ in all directions ≤−0.2 Gy (0.5%), with maximum value of −0.4 Gy (1.0%) in the A direction. Isocenter shifts of 5 mm for TFiF perturbed plans produced mean absolute difference ΔD_95_ and ΔD_98_ of −0.3 Gy (−0.7%), and −0.4 Gy (−1.0%), respectively, with maximum differences of −0.5 Gy (−1.2%) and −0.6 Gy (−1.5%), respectively, in the A direction; for VMAT perturbed plans, larger mean ΔD_95_ and ΔD_98_ values were registered: −1.5 Gy (−3.7%), and −2.0 Gy (−5.0%), respectively, with maximum differences in the A direction of −2.6 Gy (−6.3%) and 3.2 Gy (7.9%), respectively. For 10 mm isocenter shifts, mean ΔD_95_ and ΔD_98_ of −1.3 Gy (−3.2%) and −2.1 Gy (−5.2%) respectively, were registered for TFiF perturbed plans, whereas VMAT perturbed plans produced larger values of −3.8 Gy (−9.3%) and −5.6 Gy (−13.9%), for ΔD_95_ and ΔD_98_, respectively. A and R directions contributed most to worsening the target dosimetry with ΔD_95_ of −2.6 Gy (−6.4%), and −2.2 Gy (−5.4%), respectively, for TFiF plans, and −7.3 Gy (17.8%) and −6.3 Gy (−15.4%) for VMAT plans; similarly, for ΔD_98_ differences of −3.8 Gy (9.4%), and −3.2 Gy (7.9%), in the A and R direction, respectively, were obtained for TFiF plans, whereas −10.1 Gy (25.1%), and −8.9 Gy (22.1%), respectively, were reported for VMAT plans.

**Figure 1:**
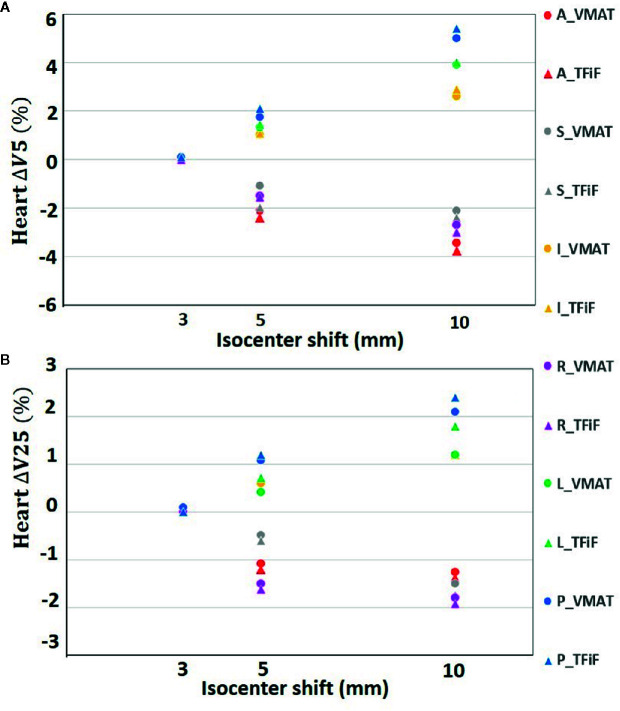
Heart V_5_ **(A)** and V_25_ **(B)** absolute differences between VMAT and TFiF references and corresponding perturbed plans, for different isocenter shifts directions and magnitudes. VMAT, volumetric modulated arc therapy; TFiF, tangential field in field; A, anterior; P, posterior; I, inferior; S, superior; R, right; L, left.

**Figure 2:**
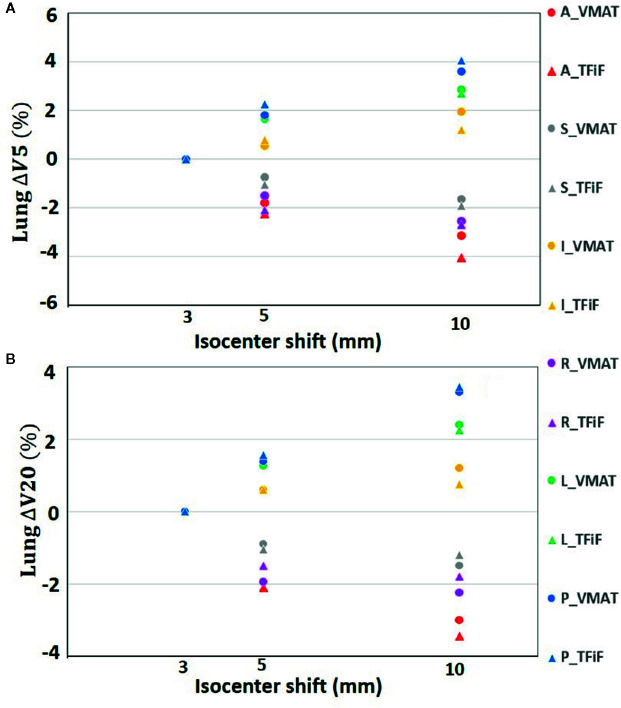
Left lung V_5_ **(A)**, and V_20_ **(B)** absolute difference between VMAT and TFiF references and corresponding perturbed plans, for different isocenter shifts directions and magnitude. VMAT, volumetric modulated arc therapy; TFiF, tangential field in field; ant, anterior; sup, superior; inf, inferior; post, posterior.

Only isocenter shifts in the L, P, and I directions increased the dose to OARs. Shifts in the other three directions (R, A, S) had the effect of moving the OARs further away from the treatment field, thus decreasing the dose received. In **Figure 1** the heart ΔV_5_ and ΔV_25_ and in **Figure 2** the lung ΔV_5_ and ΔV_20_ are plotted for each isocenter shift direction and magnitude. The mean and range values of the absolute differences for the DVH parameters between the reference plans and the perturbed plans along the L, P, and I directions are reported in **Table 4** for the heart, the left lung, and the right breast. The mean absolute difference across all OARs, treatment techniques, and perturbation values remain <2%, with the exception of the heart and left lung ΔV_5_, which rises for isocenter shifts of 10 mm to 3.9 and 2.7%, respectively, for TFiF, and 4.1 and 3.0%, respectively, for VMAT plans.

**Table 4:** Heart and lung dose volume histogram (DVH) endpoints mean and range of the absolute difference between the reference and perturbed VMAT and TFiF plans for the directions (left, posterior, and inferior) that contribute to an increase in the OAR dose.

				10 mm		5 mm		3 mm	
				VMAT	TFiF	VMAT	TFiF	VMAT	TFiF
Heart		ΔV_5_ (%)		**4.1 (2.9–5.5)**	**3.9 (3.0–4.7)**	1.5 (2.0–4.1)	1.4 (1.1–2.0)	0.1 (0.0–0.2)	0.0 (0.0–0.1)
		ΔV_10_ (%)		1.9 (1.7–34.4)	1.9 (1.5–3.3)	1.3 (1.0–2.0)	1.3 (1.0–1.8)	0.1 (0.0–0.3)	0.1 (0.0–0.3)
		ΔV_25_ (%)		1.8 (1.6–2.5)	1.5 (1.3–2.3)	0.9 (0.7–1.4)	0.7 (0.4–1.5)	0.0 (0.0–0.2)	0.1 (0.1–0.2)
		ΔD_m_ (%)		0.5 (0.7–1.3)	0.5 (0.8–1.4)	0.4 (0.4–0.6)	0.2 (0.1–0.4)	0.1 (0.0–0.3)	0.0 (0.0–0.1)
Left lung		ΔV_5_ (%)		**3.0 (2.4–5.6)**	2.7 (2.3–4.6)	1.6 (1.2–2.5)	1.3 (1.0–2.6)	0.2 (0.2–0.6)	0.2 (0.1–0.3)
		ΔV_20_ (%)		1.9 (1.7–4.0)	1.8 (1.4–3.5)	1.2 (1.0–2.3)	1.0 (0.7–1.9)	0.2 (0.1–0.4)	0.1 (0.2–0.3)
		ΔD_m_ (%)		0.8 (0.6–1.4)	0.8 (0.5–1.4)	0.5 (0.4–1.0)	0.5 (0.3–0.9)	0.4 (0.3–0.5)	0.4 (0.3–0.4)
Right breast		ΔV_5_ (%)		0.1 (0.0–0.1)	0.1 (0.0–0.1)	0.0 (0.0–0.0)	0.0 (0.0–0.1)	0.0 (0.0–0.0)	0.0 (0.0–0.1)
		ΔD_m_ (Gy)		0.1 (0.0–0.2)	0.1 (0.0–0.1)	0.0 (0.0–0.1)	0.0 (0.0–0.1)	0.0 (0.0–0.1)	0.0 (0.0–0.1)

VMAT, volumetric modulated arc therapy; TFiF, tangential field in field; D_x_, dose in Gy received by x% of the volume; D_m_, mean dose; V_x_, volume receiving x Gy.

In gray data with mean difference ≤ 2%, in bold >3%.

## Discussion

There is now sufficient evidence that DIBH in left-sided breast radiotherapy allows for dosimetric sparing of OARs (5–8). Historically, treatment plans of the breast were performed with opposing tangent fields, subsequently optimized with the field-in-field technique. In our institution, DIBH TFiF is offered to every left-sided breast patient undergoing a radiotherapy treatment after conservative surgery. The availability of a new LINAC with high dose rate drove us to consider using VMAT for these treatments and to compare them with our standard of care (TFiF). Furthermore, the idea was also to compare the response of these two irradiation techniques when perturbed by incorrect isocenter positioning, verifying whether using them in BH could have a minor effect on the dosimetric impact on the organs at risk. Among the patients studied, the BMIs were almost all within the standard level value, correctly representing the female population afferent to our hospital; in any case, BMI, heart, and total lung volumes have been reported in the literature as having a minimal impact on the target dose and lung dosimetry (38).

Both the reference TFiF and the VMAT plans provided adequate and similar CTV dose coverage (*p* > 0.05) and OAR sparing (*p* > 0.05), except for the low doses (V_5_) for which higher values were obtained in VMAT plans (*p* > 0.05). The low-dose bath exposure of healthy structures is a well-known limitation of VMAT in breast cancer treatment (39); nevertheless, the difference was small, the values obtained were well above the OAR constraints, and the VMAT plans were considered clinically acceptable. These results are in agreement with previously published studies (40).

When perturbations were introduced, however, TFiF techniques guaranteed an accurate target coverage for isocenter shifts up to 5 mm with deviations of the target DVH dosimetric parameters <1.0%, whereas VMAT plans seemed more sensitive to positioning errors registering mean deviations of −3.7% and −5.0% for D_95_ and D_98_, respectively. In **Table 3** it is possible to observe that each isocenter perturbation has a dosimetric impact on the CTV, which is larger for VMAT plans than for TFiF plans, and this is amplified for isocenter’ perturbations of 10 mm, for which mean ΔD_95_ of −9.3 *vs*. −3.2% respectively, and mean ΔD_98_ of −13.9 *vs*. −5.2%, respectively, were registered. Moreover, perturbations in the A and R directions most affected the target dosimetry for both techniques. Nevertheless, for VMAT plans perturbed with isocenter shifts of 10 mm, all the directions have a significant impact on the plan dosimetry, contributing to D_95_ deviations between −3.4% (in the I direction), and −17.8% (in the A direction); larger deviations, between −6.7, and −25.1%, in the I and A directions, respectively) were registered for D_98_. These high deviations on VMAT plans target dosimetry reported for each direction of the perturbation make a customized solution difficult. For TFiF plans, instead, mean ΔD_95_ and ΔD_98_ are smaller, and only the A and R directions, and the A, R, and I directions, respectively, contribute with deviations larger than 3%. This suggests it may be possible to mitigate the dosimetric deviations to below 3% in those specific directions for isocenter’s positioning errors of 10 mm.

For the OARs, only the L, P, and I isocenter shift directions contributed to increase the dose received, bringing the treatment field closer to the OARs. Considering only these directions that worsen the OARs dosimetry, we obtained dose values with deviations of less than 2% from the reference plan doses, with the exception of 10 mm perturbation, for which mean differences between 3.7 and 4.1% were registered for the low doses. Left sided-breast BH irradiation has therefore the advantage not only of limiting the dose to the heart and lung if compared with free breathing delivery, but also of being more robust against possible heart and lung overdosage in case of unexpected isocenter misplacement. The benefit of post-operative RT for breast cancer patients, in term of reduced risk of recurrence, has been demonstrated (41, 42); nevertheless it is well known that concurrent heart irradiation leads to an increased risk of heart disease (43), with evidence of increased risk of death of 3% per Gy of the D_m_ (44). Recent literature (45) reports no correlations between incidental heart irradiation and cardiac mortality, nevertheless there is no consensus yet on cardiac irradiation induced mortality in breast radiotherapy. Moreover, the well-known second cancer risk for contralateral breast and lung (46) force us to monitor the treatment delivery to ensure that the OAR delivered dose corresponds to the planned ones. One possible application would be in systems such as surface imaging, often used to detect setup errors, which offer real-time monitoring and beam delivery interruption if patient’s positions exceed their tolerance limits (11). Personalized thresholds in the different directions based on the results presented here would ensure accurate dose delivery.

Among this study’s limitations, it is important to highlight that a single institution was involved and the results were obtained according to our center’s working protocols. The study is focused on early breast cancer patients performing radiotherapy without an implanted tissue expander, further investigations are needed for this case. Contouring of the regions of interest and approval of treatment plans were performed by a single experienced radiation oncologist for consistency in comparing the different treatment modalities.

Jensen et al. (47) analyzed the influence of localization errors on VMAT and 3DCRT breast plans using weekly offline imaging and throughout the treatment session; their findings, representative of the workflow of their Center, show that perturbed dose calculated on the treatment data were less variable for VMAT than for 3DCRT plans. Personalized radiotherapy means being able to adapt the clinical workflow to the way the individual institute works. The knowledge and therefore the study of how the system responds to external stress is the first step towards this ambitious goal.

Left sided breast treatment performed in breath hold maintains the heart and lung doses close to the planned values (deviations < 2%) for setup errors up to 10 mm from the isocenter and for both delivery techniques, except for heart and lung low doses that increase up to 4.1%. TFiF technique guarantees an accurate target coverage for isocenter shifts of 3 mm, and 5 mm with dose deviations < 1%; for isocenter shifts of 10 mm, target dosimetric parameter deviations ranged between −3.2 and −5.2%; the main contribution to these deviations is due to perturbations in specific directions, leaving open the possibility to limit them with appropriate personalized management of the treatment. VMAT plans seem more sensitive to positioning errors, showing mean target dosimetric parameters deviations up to 5% for 5 mm isocenter shifts, and up to 13.9% for 10 mm isocenter shifts, leaving less room to control the target dosimetry. The evaluation of the dosimetric impact when the ideal system is perturbed therefore remains of primary importance. Quantitative information may support radiation oncologists in setting up a personalized radiotherapy.

## Data Availability Statement

The raw data supporting the conclusions of this article will be made available by the authors, without undue reservation.

## Ethics Statement

The studies involving human participants were reviewed and approved by Ethics Committee of Sichuan Cancer Hospital (approval number SCCHEC 02-2020003). The patients/participants provided their written informed consent to participate in this study.

## Author Contributions

Design of the research: LCO, YZ, JL. Treatment plans: DZ, JW, XX, PD, ML. Statistical Analysis: DF, JL. Manuscript preparation: DF, XX, JW. Manuscript writing: LCO, YZ. Manuscript final revision: DF, DP, JL. All authors contributed to the article and approved the submitted version.

## Funding

This research was supported by Chengdu Science and Technology Project (2019-YF09-00095-SN).

## Conflict of Interest

The authors declare that the research was conducted in the absence of any commercial or financial relationships that could be construed as a potential conflict of interest.

The handling editor declared a past co-authorship with one of the authors DF.

## Abbreviations

DIBH, deep inspiration breath hold; TFiF, tangential field in field; MLC, multi leaf collimator; VMAT, volumetric modulated arc therapy; OARs, organs at risk; CT, computed tomography; CTV, clinical target volume; PTV, planning target volume; D_m_, mean dose; DVH, dose volume histogram.
